# Toxicological evaluation of convulsant and anticonvulsant drugs in human induced pluripotent stem cell-derived cortical neuronal networks using an MEA system

**DOI:** 10.1038/s41598-018-28835-7

**Published:** 2018-07-10

**Authors:** A. Odawara, N. Matsuda, Y. Ishibashi, R. Yokoi, I. Suzuki

**Affiliations:** 1 Department of Electronics, Graduate School of Engineering, Tohoku Institute of Technology, 35-1 Yagiyama Kasumicho, Taihaku-ku, Sendai, Miyagi 982-8577 Japan; 2 iPS-non-Clinical Experiments for Nervous System (iNCENS) Project, Kanagawa, Japan; 3 Consortium for Safety Assessment using Human iPS Cells (CSAHi), Kanagawa, Japan; 4 Advanced Institute for Materials Research, Tohoku University, 2-1-1 Katahira, Aoba-ku, Sendai, Miyagi 982-8577 Japan; 5 Japan Society for the Promotion of Science, Tokyo, Japan

## Abstract

Functional evaluation assays using human induced pluripotent stem cell (hiPSC)-derived neurons can predict the convulsion toxicity of new drugs and the neurological effects of antiepileptic drugs. However, differences in responsiveness depending on convulsant type and antiepileptic drugs, and an evaluation index capable of comparing *in vitro* responses with *in vivo* responses are not well known. We observed the difference in synchronized burst patterns in the epileptiform activities induced by pentylentetrazole (PTZ) and 4-aminopryridine (4-AP) with different action mechanisms using multi-electrode arrays (MEAs); we also observed that 100 µM of the antiepileptic drug phenytoin suppressed epileptiform activities induced by PTZ, but increased those induced by 4-AP. To compare *in vitro* results with *in vivo* convulsive responses, frequency analysis of below 250 Hz, excluding the spike component, was performed. The *in vivo* convulsive firing enhancement of the high γ wave and β wave component were observed remarkably in *in vitro* hiPSC-derived neurons with astrocytes in co-culture. MEA measurement of hiPSC-derived neurons in co-culture with astrocytes and our analysis methods, including frequency analysis, appear effective for predicting convulsion toxicity, side effects, and their mechanism of action as well as the comparison of convulsions induced *in vivo*.

## Introduction

Human iPSC-derived neurons are used to evaluate toxicity to the human nervous system, and are expected to be applied to toxicity evaluations in nonclinical studies^1,2^. Assay systems using these neurons include differentiation, neurite growth, synaptogenesis, neuronal network formation, and functions for toxicity evaluation^3,4^. One of the major toxicities of the central nervous system in clinical trials is convulsions^5^. The evaluation of the potential for convulsions requires an assay system that can measure functions. Multi-electrode array (MEA) systems have recently attracted attention as useful for evaluating convulsions because they can non-invasively measure the electrophysiological activities of neural networks^6–9^. Indeed, MEAs have been successfully used to evaluate drug responses in iPSC-derived cardiomyocytes for the screening of compounds with QT prolongation and proarrhythmic potential^10,11^. We have also previously reported the effectiveness of MEA measurements for drug responses in human iPSC-derived neurons^6,12,13^, which has since been confirmed by several other groups^7,14–16^.

In the case of human iPSC-derived neurons, it takes time to bring the cells to maturation, so it is necessary to identify a culture period suitable for the functional evaluation of drug responses^6,12,14^. We and other groups have shown that the astrocyte co-culture method is effective as a culturing method to promote maturation^6,7,12,17^. However, to aim for a more practical application, early maturation is required, and the difference in responsiveness between astrocytes and neurons alone has not been sufficiently investigated for convulsive compounds. In addition, the differences in responsiveness of MEAs due to varying convulsive compounds are not known.

The evaluation of antiepileptic drugs using human iPSC-derived neurons is also an effective evaluation system for drug discovery development and side effect detection. It is important to be able to detect the positive effects of antiepileptic drugs (AEDs), but as the mechanisms of action of antiepileptic drugs are complex, the detection of side effects is equally important. For example, it has been reported that side effects occur with the classical antiepileptic drug phenytoin^18–23^.

An important issue in constructing an *in vitro* evaluation system using human iPS C-derived neurons is how *in vivo* responses can be predicted from *in vitro* responses. In order to address this obstacle, it is necessary to compare *in vitro* responses with *in vivo* responses. We have focused on the frequency analysis of the local field potential (LFP) during epilepsy acquired in *in vivo* human electrocorticogram (ECoG)^24–31^. Since the data of MEA is also LFP, we considered that the comparison of frequency characteristics was possible. Studies analyzing human ECoG and electroencephalogram (EEG) *in vivo* data have reported that high gamma and beta wave components are enhanced in epilepsy^24–32^. Analysis of MEA in cultured neural networks has been detected by analyzing spikes with high frequency components of 1 kHz or more; however, we analyzed low frequency components up to 250 Hz as discussed in *in vivo* LFP analysis. We attempted to investigate whether the enhancement phenomena of high γ wave or β wave components are detected during epileptiform activities as detected *in vitro* by MEA.

In this study, we investigated differences in the responsiveness between Pentylenetetrazol (PTZ, GABA_A_ antagonist) and 4-Aminopyridine (4-AP, K^+^ channel antagonist), which are typical seizure-inducing drugs with different mechanisms of action. We used both neuronal and astrocyte co-cultured samples, and investigated the effect of AEDs by administering Phenytoin upon convulsive-like ignition. In addition, frequency analysis by the administration of PTZ and 4-AP was performed to investigate whether changes observed during *in vivo* human epilepsy were detected, and whether there was a difference in frequency characteristics between PTZ and 4-AP administration.

## Results

### Spontaneous firing in hiPSC-derived cortical neurons and co-culture with astrocytes

**Figure 1:**
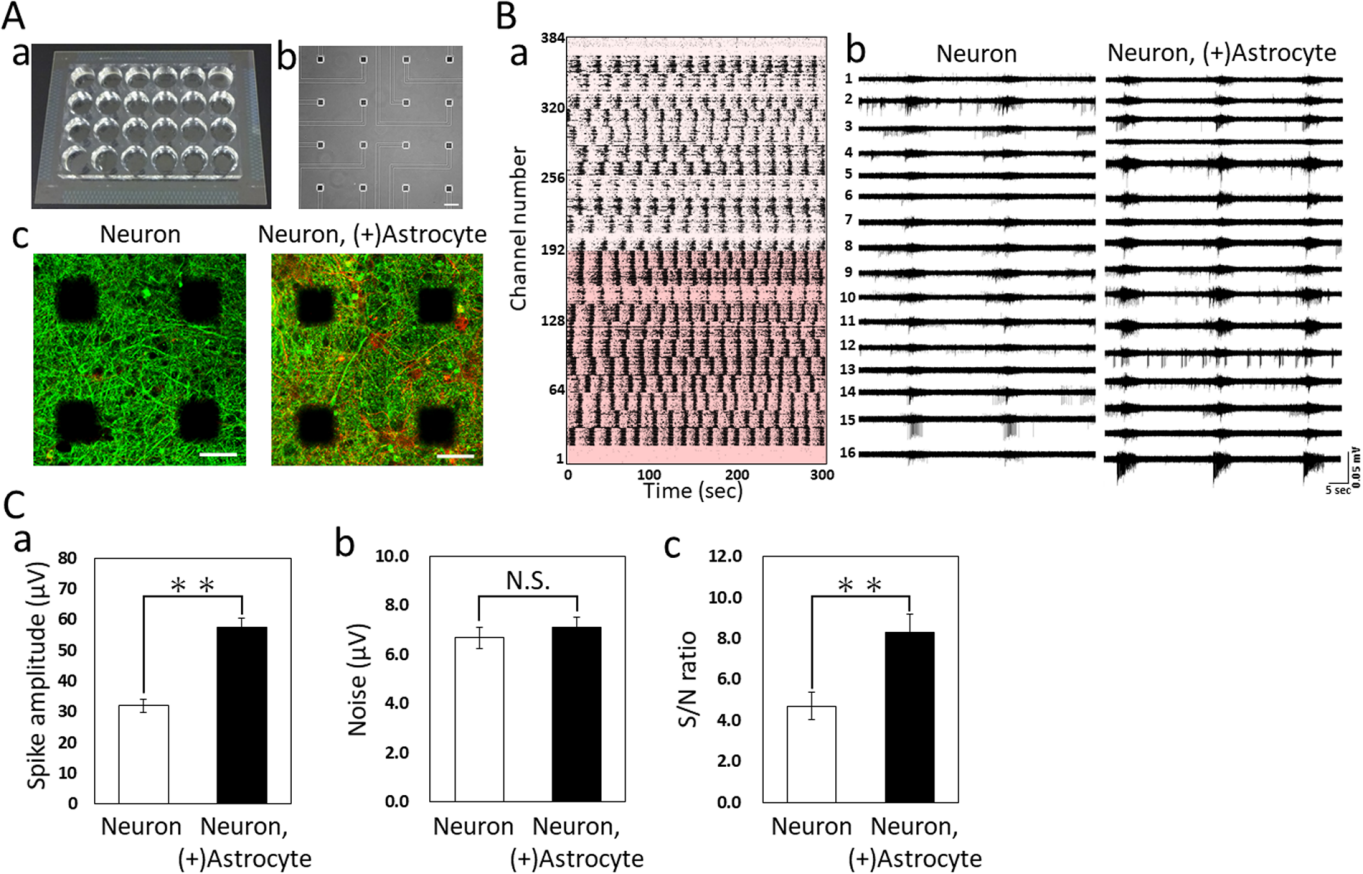
Extracellular recordings of spontaneous firing in hiPSC-derived cortical neurons and co-culture with hiPSC-derived astrocytes using a 24-well MEA system. (**A**) 24-well MEA plate. (a) Overview of an MEA plate. (b) Phase contrast image of 16 electrodes per well. Scale bars = 100 μm. (c) Immunofluorescent image of neurons and co-culture with astrocytes on an MEA chip at 11 weeks *in vitro* (WIV). Images show the neurons using β-tubulin III (green) and astrocytes using GFAP (red) immunostaining. Scale bars = 50 μm. (**B**) Typical spontaneous firing patterns at 8 WIV. (a) Raster plots of spontaneous firing for 5 min at 384 electrodes of 24 wells. Co-culture with astrocyte samples show below 192 electrodes. Raster plots above 192 electrodes is the data of neuron samples. (b) Typical waveform of synchronized burst firings (SBFs) for 1 min at 16 electrodes per wells in neurons (left) and co-culture with astrocytes (right). (**C**) Signal to noise ratio in the neurons only culture sample and co-culture sample. (n = 5 wells, two-tailed paired Student’s *t*-test, **p < 0.01) (a) Spike amplitude in neurons and co-culture sample. Spikes with negative voltage were detected in spontaneous firings for 10 min. The spike amplitude was calculated as an absolute value. (b) Noise in neurons and co-culture samples. Noise was calculated as the |average + S.D.| + |average − S.D.| of the voltage without spikes for 1 second. (c) The signal to noise ratio was calculated by dividing the average of spike amplitude by noise for each well.

**Figure 2:**
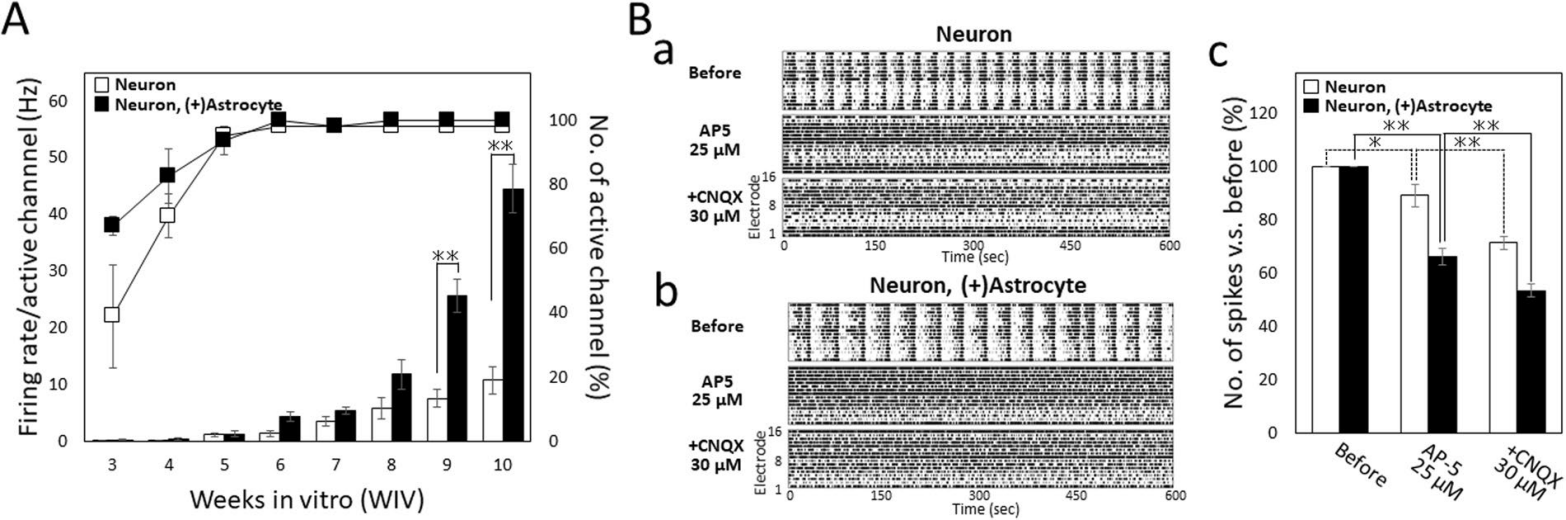
Development of spontaneous firings and functional maturation. (**A**) Firing rate per active electrodes of neurons and co-culture with astrocytes (average for 10 min) from 3 to 10 WIV. White bar and black are neurons and co-culture, respectively. Differences in the firing rate between the neurons and co-culture samples were analyzed using two-way ANOVA followed by the Holm–Bonferroni Method. (**p < 0.001). Line graph shows the percentage of the number of active electrodes per 16 electrodes. n = 4 wells. (**B**) Electrophysiological function of glutamatergic receptors. (a) Raster plots of spontaneous firings in neuron samples for 5 min before, 25 µM AP-5, and cumulative 30 µM CNQX administration. (b) Raster plots in co-culture sample. (c) Change in total number of spikes before (100%, baseline) and after AP-5, and CNQX administration in neurons (white) and co-culture (black). (neurons, n = 4 wells, co-culture, n = 10 wells, one-way ANOVA and post hoc Dunnett’s test, *p < 0.05, **p < 0.01).

To investigate the functional maturation over time in culture, and the difference between only neurons in culture and co-culture with astrocytes, we established co-cultures of hiPSC-derived cortical neurons and astrocytes on 24-well MEA chips (Fig. 1A–a) and recorded spontaneous firings once every week. Figure 1A–a and b show the 24-well MEA chips and the phase contrast image of 16 electrodes of one well, respectively. Human iPSC-derived cortical neurons and co-cultures with astrocytes grown on MEA chips survived over the long-term without cell aggregation (Fig. 1A–c and b), enabling the measurement of distributed network field activity. Representative examples of spontaneous firing patterns at 8 weeks *in vitro* (WIV) revealed high detection rate of spontaneous firings in the 24 wells and electrodes per well (Fig. 1B–a). Spontaneous firings were detected at almost all channels per well both with neurons alone and co-culture samples (Fig. 1B–b). The signal to noise ratio and the number of firings in co-culture samples were higher than the neurons only culture samples. The spike amplitude in the neurons only sample was 32 ± 2.2 µV, whereas that of the co-culture sample was 58 ± 2.8 μV (Fig. 1C–a). The noise level was 6.7 ± 0.4 µV in the neurons only sample and 7.1 ± 0.4 µV in the co-culture sample; there was no significant difference between the samples (Fig. 1C–b). The signal to noise ratio was 4.7 ± 0.3 in the neurons only sample, whereas in the co-culture sample, it was 8.3 ± 0.4, which was significantly higher compared with that in the neuron only sample (Fig. 1C–c). Spontaneous firings were first detected at 3 weeks of culture and increased with culture time. Synchronized burst firings (SBFs), wherein multiple neurons in neuronal networks fire within tens of milliseconds to several seconds because of synapse propagation, were detected from 5 WIV in the co-culture samples and 6 WIV in the neuron alone samples. SBF indicates functional maturation in synaptic transmission. Spontaneous firing rates significantly increased depending on the weeks of culture, and the firing rate in the co-cultured samples were higher than that of the neuron alone sample (two-way ANOVA, P < 0.001). A significant difference in firing rates per channel between the neuron only and co-culture sample was observed at 9 and 10 WIV (Fig. 2A–a, Holm–Bonferroni method, **P < 0.01). Firing rates per channel in co-culture exceeded 40 Hz at 10 WIV more than thrice in the neuron only sample. Although the active electrodes, where a spike was detected, in the co-culture were higher than the neurons alone at the 3 and 4 WIV, the active electrodes reached 100% at the 6 WIV in both culture samples.

### Functional maturation of hiPSC-derived cortical neurons and co-culture with astrocytes

To evaluate the maturation of synaptic function, we measured changes in spontaneous firing following the application of the AMPA/kainate GluR antagonist CNQX (30 μM), or the NMDA GluR antagonist AP-5 (25 μM) at 6 WIV (Fig. 2B). The electrophysiological responses of NMDA receptors are one of the indicators of the maturation of synaptic function. In 6 WIV cultures, AP-5 lost SBFs both in the neurons and co-culture samples, and decreased the firing rates to 89.1% ± 4.21% and 66.0% ± 3.13% before administration in co-culture and in neurons, respectively. Firing rates were further decreased to 71.5% ± 2.52 and 53.5% ± 2.56%, respectively, by the cumulative administration of CNQX (Fig. 2C). These results indicated that NMDA receptors contribute to SBF at 6 WIV both in neurons and co-culture samples, and that AMPA receptors also function electrophysiologically. The decrease in the firing rate by glutamate receptor inhibition was significantly detected in the co-culture sample compared with in the neuron only sample (one-way ANOVA, P < 0.001). The neural network recovers after washout, and on the next day, SBFs can be seen and drug testing is possible in both samples (Supplementary Fig. 1). To confirm synaptogenesis, we performed immunochemical staining for the presynaptic marker, synaptophysin. Many synapses were formed both in the neuron only and co-culture sample on the MEA (Supplementary Fig. 2). In summary, these results indicated that both AMPA and NMDA receptors work electrophysiologically, and pharmacological tests can be performed over 6 WIV.

### Elctrophysiological responses against 4-AP and PTZ and the effects of phenytoin

To evaluate the responses of the human iPSC-derived neural networks for modeling toxicological assays, we examined the epileptiform activity against pentylentetrazole (PTZ) and 4-aminopryridine (4-AP), which are typical convulsant drugs with different action mechanisms. We also examined the anti-convulsant effects of phenytoin during cumulative administration. PTZ and 4-AP experiments were conducted at 9 WIV (67 DIV) both neurons and co-culture samples.

**Figure 3:**
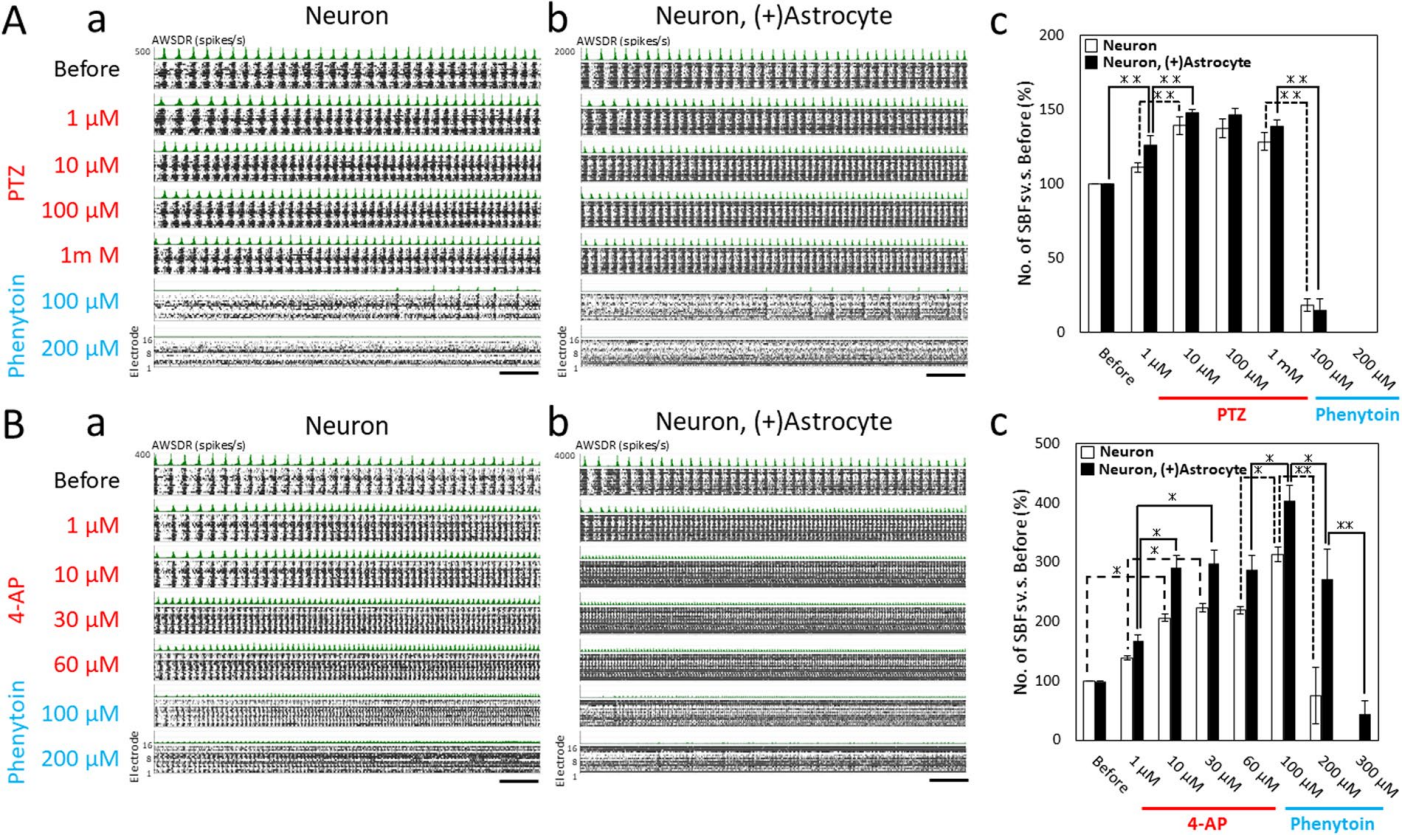
Induction of epileptiform activity by pentylentetrazole (PTZ) and 4-Aminopyridine (4-AP) administration, and the anticonvulsant effects of phenytoin at 9 WIV. Experiments of PTZ and 4-AP were performed in different wells of a 24-well MEA plate. (**A**) Induction of epileptiform activity using PTZ and the suppressive effect of phenytoin. (a) Raster plots for 1 min at PTZ and phenytoin administration in the neuron sample. PTZ was added to the culture medium at increasing concentrations (1 µM, 10 µM, 100 µM, and 1 mM). Phenytoin was then added (100 µM and 200 µM). (b) Responses of PTZ and phenytoin in co-culture with astrocytes. Scale bars = 1 min. (c) Changes in the number of SBFs versus before (%) during drug treatment in neurons and co-culture with astrocytes (n = 5 wells, **p < 0.01). Data were analyzed using one-way ANOVA followed by the Holm–Bonferroni method. (**B**) Induction of epileptiform activity using 4-AP, and the effects of phenytoin. (a) Raster plots for 1 min at 4-AP and phenytoin administration in neuron samples. 4-AP was added to the culture medium at increasing concentrations (1 µM, 10 µM, 30 µM, and 60 µM). Phenytoin was then added (100 µM, 200 µM, and 300 µM). (b) Responses of 4-AP and phenytoin in co-culture with astrocytes. Scale bars = 1 min. (c) Changes in the number of SBFs versus before (%) and during drug treatment in neurons and co-culture with astrocytes (n = 6 wells, *p < 0.05, **p < 0.01).

Figure 3Ba and b show the raster plot for all 16 electrodes, and the array-wide spike detection rate (AWSDR), in which this histogram shows the number of spikes per 1 sec detected by MEA with 1 ms bin size of neurons and the co-culture samples before, 1, 10, 100, and 1000 µM PTZ administration and 100 and 200 µM phenytoin cumulative administration. The temporal correspondence with spikes in multiple electrode channels indicates that these AWSDR events correspond to synchronized burst firings (SBFs). Pentylentetrazole induced an increase of SBFs both in neurons and co-culture samples. The number of SBFs increased to 136% ± 6.14% and 148% ± 3.02% of baseline (to 43 ± 1.9 bursts and 52 ± 1.4 bursts for 10 min) at 10 µM PTZ in neurons and co-culture samples, respectively (Fig. 3A–c). Although the number of SBFs decreased slightly to 127% ± 6.12% and 138% ± 3.05% at 1000 µM, the increase in SBFs was maintained compared with before administration. After sequential administration of 100 µM phenytoin, the SBF number dramatically reduced to 17.8% ± 4.14% and 11.9% ± 5.74% in neurons and co-culture samples, respectively. The administration of 200 µM phenytoin lost SBFs in both culture samples and reduced firing frequency dramatically compared with baseline (Fig. 3A).

The administration of 4-AP significantly increased SBFs in a concentration-dependent manner both in neurons and co-culture samples (Fig. 3Ba and b). The number of SBFs increased to 225% ± 8.44% and 298% ± 22.6% of baseline (to 77 ± 3.2 bursts and 111 ± 9.7 bursts for 10 min) at 30 µM 4-AP in neurons and co-culture samples, respectively, and high frequency SBFs were kept at 60 µM 4-AP administration (Fig. 3A–c). Interestingly, after the sequential administration of 100 µM phenytoin, SBF number increased to 318% ± 11.1% and 405% ± 26.3% (to 109 ± 4.3 bursts and 150 ± 10.5 bursts for 10 min), respectively (Fig. 3Ba,b and c, Supplementary Movie 1). This was different from the responses after PTZ and Phenytoin administration. Although high frequency SBFs in neurons at 200 µM administration were dramatically decreased at 75.1% ± 50.9%, high frequency SBFs in co-culture samples were decreased to only 273% ± 50.7%, and kept at high frequency SBFs. At 300 µM, phenytoin suppressed SBFs and reduced the total spike frequency in both culture samples (Fig. 3B–a,b and c).

Collectively, these results indicate that epileptiform activities occur with typical convulsant drugs in a cultured hiPSC-derived neuron system at 9 WIV. The rate of increase in SBFs in co-culture with astrocytes was higher than in neurons, but the trend of increase for both culture samples was the same between PTZ and 4-AP administration. Although phenytoin has anticonvulsant efficacy in both culture systems, we found that 100 µM phenytoin increased the epileptiform activities induced by 4-AP. This paradoxical result indicates the potential side effects of phenytoin, and the concentration of the antiepileptic effect of phenytoin differs between neurons and co-culture.

### Difference of synchronized burst firings between PTZ and 4-AP

**Figure 4:**
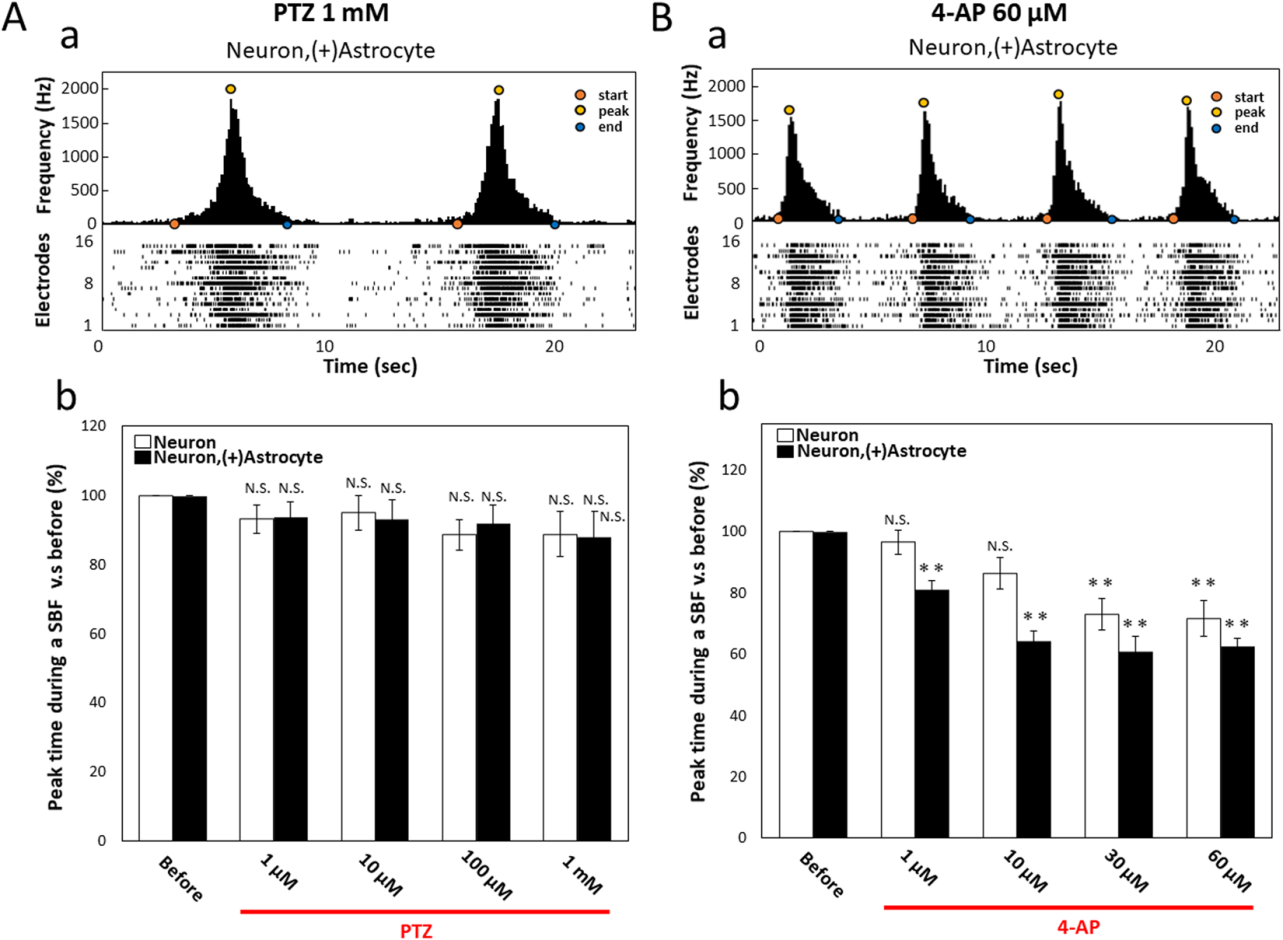
Peak time during an SBF with PTZ and 4-AP administration. (**A**) Representative peak of an SBF at PTZ 1 mM administration. (a) Upper graph shows the histogram of spikes during an SBF obtained at 16 electrodes (orange circle; start time of SBFs, yellow circle; peak time of SBFs, blue circle; end time of SBFs). Under graph shows raster plots of spontaneous firing at 16 electrodes. (b) Dose dependency in neurons and co-culture samples (n ≧ 5, N.S. is not significant). Data were analyzed using one-way ANOVA followed by post hoc Dunnett test. (**B**) Representative peak of an SBF at 4-AP 60 µM administration. (a) The peak time of spikes during an SBF shifted to the beginning of the SBF. (b) Dose dependency in neurons and co-culture samples (n ≧ 5, **p < 0.01).

To evaluate the difference of responses between PTZ and 4-AP, we analyzed the shape of the burst histogram. Figure 4A–a and B–a shows typical histograms and raster plots for the responses of 1 mM PTZ and 60 µM 4-AP administration. The peak spikes during a SBF were the middle part of the SBF and not changed by concentration-dependent PTZ administration both in neurons and co-culture samples (Fig. 4A–b). Also, no difference was observed between the neuron only and co-culture samples (one-way ANOVA, P = 0.960). However, 4-AP shifted the peak to the first part of SBFs and increased the peak of the spikes. We defined that the baseline of 100% was the period-time from the start time of the SBF to the time of the peak spikes. The peak time was shortened to 96.5% ± 3.87% at 1 µM, 86.4% ± 5.10% at 10 µM, 73.0% ± 5.21% at 30 µM, and 71.8% ± 5.87% at 60 µM in the neurons sample, and 81.1% ± 2.99% at 1 µM, 64.1% ± 3.13% at 10 µM, 60.9% ± 4.74% at 30 µM, and 62.7% ± 5.87% at 60 µM in the co-culture sample (Fig. 4B–b). Although the trend in which the peak time was shortened in a dose-dependent manner detected both in neurons and co-culture samples was the same, co-culture samples showed more pronounced changes compared with neuron samples (one-way ANOVA, P < 0.001). The peak sift of 4-AP administration obtained with 64 electrodes appeared more prominently (Supplementary Fig. 3). Differences between 4-AP and PTZ were observed even when the cell seeding date and sample were different.

### Frequency analysis in the pharmacological properties of spontaneous firing

**Figure 5:**
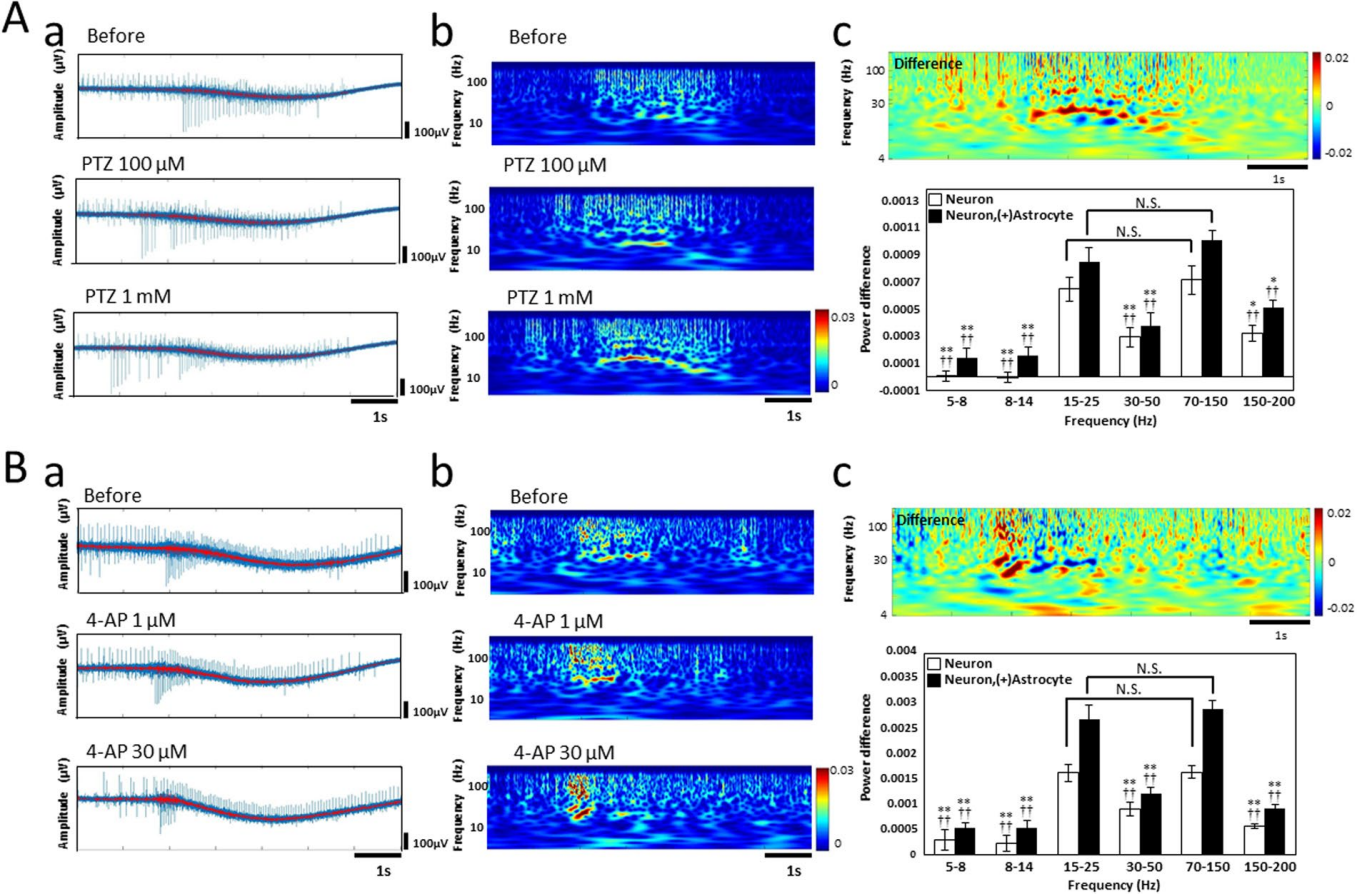
Frequency analysis of SBFs with PTZ and 4-AP administration. (**A**) Wavelet analysis of the SBF induced by PTZ. (a) The local field potential (LFP) of an SBF before, 100 µM, and 1 mM PTZ obtained by the same electrode. LFP, high-pass filtered at 1 Hz was recorded. Red line shows frequency components below 250 Hz using a FIR high cut filter. (b) Corresponding scalograms of temporal scales during the application of PTZ are shown as left traces. The scalograms are computed from the raw trace, not the high-pass filtered data. Wavelet phase coherence with its own color bar shown to the right of it. (c) Scalograms of the difference between 1 mM PTZ and before administration. The graph below the scalograms shows the positive and negative changes in each band quantified by average wavelet transform coefficient per pixel in the neurons and co-culture samples, respectively. N is the analysis data of 6 electrodes. Five SBFs per electrode were analyzed and average values were used. Electrodes with a high S/N ratio were selected, and five typical SBFs with many active electrodes were selected. Statistical analysis used the the Holm–Bonferroni method. *indicates a significant difference with respect to β wave band (15–25 Hz), and ^†^indicates a significant difference with respect to high-γwave band (70–150 Hz; (*p < 0.05, **p < 0.01, ^†^p < 0.05, ^††^p < 0.01). (**B**) Wavelet analysis of the SBF induced by 4-AP. (a) The local field potential (LFP) of an SBF before, 1 µM, and 30 µM 4-AP obtained by the same electrode. (b) Corresponding spectrograms of temporal scales during the application of 4-AP are shown as left traces. (c) Spectrograms of the difference between 30 µM 4-AP and before administration. The graph below the scalograms shows the positive and negative changes in each band quantified by average wavelet transform coefficient per pixel in neurons and co-culture samples, respectively. (n = 6 electrodes, 5 SBFs were analyzed per electrode. *is versus 15–25 Hz. ^†^is versus 70–150 Hz. *p < 0.05, **p < 0.01, ^†^p < 0.05, ^††^p < 0.01).

To evaluate the difference of responses between PTZ and 4-AP administration, and to compare the epileptiform activities between *in vivo* human brain and *in vitro* human iPSC-derived neurons, we focused on the frequency analysis of SBFs from the raw data. Figure 5A–a and b shows the LFPs and wavelet analysis of SBFs at before, 100 µM, and 1 mM PTZ administration. Red waveforms in LFP show the waveform data below 250 Hz, excluding spike components (Fig. 5A–a). Wavelet analysis showed that β wave component (15–25 Hz) and high γ component (70–150 Hz) increased dose-dependently (Fig. 5A–b). The time during which the frequency component strengthened was the middle part of the SBF, similar to the results of the peak time in Fig. 4. Figure 5A–c showed the differential value of the wavelet analysis before and after 1 mM PTZ administration. At each time in the burst, red indicates the enhancement of the frequency component, while blue indicates a decrease. The graph in Fig. 5A–c shows the quantitative data of frequency component changes between before and after 1 mM PTZ administration in neurons and co-culture samples, respectively. The frequency components of the β wave and the high γ wave were significantly intensified compared with other frequency bands in both the neurons and co-culture samples (*p < 0.05, **p < 0.01, ^††^p < 0.01). Figure 5Ba and b shows the LFPs and wavelet analysis of SBFs at before and after 1 µM and 30 µM 4-AP administration. In 4-AP administration, β waves and high γ wave components also were strengthened more than with PTZ. Interestingly, the enhancement of the frequency component shifted concentration-dependently to the beginning of burst firing (Fig. 5B–b). The differential value of the wavelet analysis before and after 30 µM 4-AP administration indicated a strong shift to the first half of the burst firing of the β wave and the high γ wave components (Fig. 5B–b,B–c). The β wave and high γ wave were also significantly intensified compared with other frequency bands both neurons and co-culture samples (**p < 0.01, ^††^p < 0.01). The phenomena of β waves and high γ waves being enhanced by PTZ and 4-AP were confirmed in other MEA dish as well (Supplementary Fig. 4) . Although the strength of the β wave and high γ wave component was also detected in both culture samples, the enhancement of frequency components in the co-culture sample tended to be larger than that in the neurons sample (Fig. 5A–c,B–c, and Supplementary Fig. 4).

In the low frequency component (not the spike component), we succeeded in detecting the frequency characteristic that can be compared with the ECoG. We found that PTZ and 4-AP enhanced β-wave and high γ-wave components in a dose-dependent manner. In 4-AP and PTZ, it was found that the time-period during which the frequency was strengthened differed. These results indicated that frequency analysis is effective for classifying the action mechanisms and frequency analysis *in vitro* and human iPSC-derived neurons MEA data makes it possible to compare with *in vivo* human ECoG data.

## Discussion

We detected synchronous burst firing at 5 to 6 weeks of culture at 100% active electrodes, and the frequency per electrode at 10 weeks of astrocyte co-culture exceeded 40 Hz. These results were obtained by using a specific cultivation protocol, including the utilization of BrainPhys medium suitable for electrophysiological maturation^33^, cell density, and coating conditions, and the utilization of 24-well MEAs with high sensitivity. The electrophysiological responses of synchronized bursts and NMDA receptors, which are indicators of functional maturation, were observed in the 6 WIV of relatively early cultivation, which is effective for conducting pharmacological tests. We previously reported that spontaneous activity is activated by the co-culture of human iPSC-derived neurons and rat astrocytes^6,12^. In this experiment, we also found that co-culture with human iPSC-derived astrocytes enhances the spontaneous activity of neuronal networks.

If we can predict the convulsion toxicity of medicines from MEA data, and predict the mechanisms of action from epileptiform activities, this should prove to be an effective assay for toxicity evaluation and drug discovery development. In these experiments, we detected the epileptiform activities induced by PTZ, a GABA_A_ blocker, and by 4-AP, a K^+^ channel blocker.

Epileptiform activities due to the increased number of SBFs were detected in both PTZ and 4-AP administration. As a result of focusing on the peak time in the histogram of the synchronous burst, we found that PTZ did not change the time compared with before administration; however, 4-AP did change the time of the peak position and maximum peak firing occurred immediately after the occurrence of the SBFs. The result of PTZ showing a peak in the middle of the SBFs is considered to be caused by the recurrent excitation propagation due to the inhibition of the GABA_A_ receptor. On the other hand, the result of 4-AP, which showed a peak at the early stage of the SBFs, is considered to be as a result of the membrane potential rising due to the block of the K^+^ channel, and each cell generated burst firing simultaneously at the first synaptic transmission. These results suggest that the peak time analysis in SBFs is effective as one analytical method to separate the mechanisms of action in epileptiform activities.

If you can find the action of AEDs in the MEA system, it will be effective for the development of AEDs and the detection of side effects. In this study, Phenytoin was administered cumulatively to detect the effects of AEDs after convulsion induction by PTZ and 4-AP. In PTZ administration, SBFs disappeared dose-dependently, but SBFs increased more than 300% compared to baseline with 100 μM phenytoin administration in the presence of 60 µM 4-AP (Fig. 3B). The mechanism of action of4-AP involves the inhibition of K^+^ channels, increase the resting membrane potential^34,35^, and increasing the permeability of Na + and Ca^2+^ channels and the release of glutamate^36–38^. Although the action of phenytoin is Na + channels and Ca^2+^ channels block^39,40^, simulation studies in silico have reported that firing increases when phenytoin is administered at high resting membrane potential^41^. The cause of our results may be the reduction of calcium entry by phenytoin administration and the consequently reduced activation of calcium-activated potassium channels. Ca^2+^ activated K^+^ channels work to decrease the resting membrane potential^42^. We considered that the increased epileptiform activities by phenytoin administration in the presence of 4-AP were caused by a further increase in resting membrane potential due to the inhibition of Ca^2+^ activated K^+^ channels. In *in vivo* EEG experiments, it has been reported that epileptiform EEG activity induced by 4-AP is suppressed in the presence of high concentrations of phenytoin (25 mg/kg). Epileptiform activity was not prevented at low concentrations of phenytoin (2.5 mg/kg) and increased latency in min to epileptiform activity was observed^43^. Our *in vitro* results indicated a firing rate decrease at high concentrations of 300 µM phenytoin, this phenomenon was consistent with *in vivo* results. Although there have been reports that activities are decreased with Na^+^ channel blockers like phenytoin administration in *in vitro* evaluation systems^39^, there has been no report showing convulsive ignition *in vitro* by Na^+^ channel blockers. Our report is the first case in which convulsions were induced *in vitro* with a Na^+^ channel blocker. These results show the possibility for reproducing the *in vivo* phenomenon and analyzing mechanisms of paradoxical and unpredictable convulsions induced by Na^+^ channel blockers.

Finding the association between *in vitro* and *in vivo* convulsive firing is extremely important for constructing a convulsion prediction system *in vitro*. In order to make *in vivo* and *in vitro* data comparison possible, we analyzed the low frequency band below 250 Hz from human ECoG and EEG data. For both PTZ and 4-AP, it was observed that beta wave and high gamma wave components were dose-dependently enhanced. The major phenomenon that the high γ component is enhanced during epilepsy has been reported from human ECoG and EEG data^25–29,32^. Enhancement of the β wave component has also been reported^25–28,32^. A similar phenomenon was also observed in our *in vitro* MEA measurements in human iPSC-derived neurons. ECoG and EEG measurements mainly acquire *field* post synaptic potentials generated from the cerebral cortex. Frequency analysis of *in vitro* MEA is also considered detection of a change in *field* post synaptic potential because it is a low frequency analysis lacking the spike component. Therefore, we consider that these *in vitro* results can be compared with *in vivo* ECoG data using the same *field* post synaptic potential information. Because EEG is affected by myoelectricity, ECoG is preferable for comparison. *In vivo* ECoG data is the SBF in a column with a 6-layer structure, whereas *in vitro* MEA data is the SBF in random 2-dimentional neuronal networks. Although the structure differs, *in vivo* ECoG and *in vitro* MEA similarly detect the response of the local circuit, and the measurement samples used are the same human cortical neurons. *In vitro* and *in vivo* cortical neuronal networks and changes in *field* post synaptic potentials in SBFs during convulsions may have similar properties. We expected enhancement of the frequency component to be an effective parameter for predicting *in vivo* convulsions. We also found differences between 4-AP and PTZ by frequency analysis. A strong frequency component was detected dose-dependently in the mid-SBFs in PTZ, and in the first half of SBFs in 4-AP. Since differences were observed at low frequencies, excluding the spike components, these results suggest that there is a difference in the synaptic current in the SBFs. It is suggested that the frequency analysis of MEA is also effective for the separation of action mechanisms. This is the first report of frequency analysis excluding spike components from MEA measurement data (LFP) of cultured neural networks *in vitro*. This frequency analysis is a frequency band from which the main action potential component has been removed, and it is considered to reflect the information of the synaptic current. If the synapse information can be used as a parameter in *in vitro* MEA measurements of cultured neural networks, it becomes a new evaluation system and the improvement of accuracy in drug evaluation is expected.

The difference in spontaneous firing frequency between neurons and co-cultures with astrocytes increased with culture days. The responses to PTZ and 4-AP between neurons and co-cultures with astrocytes showed the same trend in the case of SBFs analysis with 100% before administration, but the absolute values were significantly different. In addition, epileptiform ignition in phenytoin administration was markedly different from co-culture samples, and the disappearing concentration also differed. In frequency analysis, there was a significant difference between co-culture with astrocytes and neurons alone, and a stronger frequency change was detected in the co-culture samples. Since co-culture samples showed similar results to *in vivo* human ECoG data, co-culture samples with astrocytes are considered to be suitable for *in vivo* toxicity prediction.

In summary, time-course changes in the spontaneous activity of human iPSC-derived cortical neurons and co-culture with astrocytes samples were measured using 24-well MEA with a high S/N ratio, and the electrophysiological function associated with synaptic transmission was confirmed at 6 weeks of culture. We found that the number of SBFs increased in neurons and astrocyte co-cultured samples with administration of 4-AP and PTZ, which are typical convulsants, and we found differences in induced SBFs between PTZ and 4-AP. We first detected the paradoxical effect of phenytoin, which increased epileptiform activities in the presence of 4-AP. Frequency analysis of MEA showed that the increase of the high γ wave and β wave observed in the *in vivo* human brain during epilepsy were also detected in *in vitro* human iPSC-derived neurons after PTZ and 4-AP administration. This study shows that astrocyte co-culture is close to the *in vivo* response compared with neurons alone, and the frequency analysis method is effective for the comparison with human *in vivo* data and to separate action mechanisms. Collectively, our findings suggest that our culture and measurement and analysis protocol is effective as a toxicity evaluation system for the human nervous system *in vitro*.

## Materials and Methods

### Culture of hiPSC-derived cortical neurons

Human induced PSC-derived cortical neurons^44^ (XCL-1, XCell Science Inc., USA) were cultured at 3.0 × 10^5^ cells/cm^2^ on 16-channels per well across 24-well MEA plates (Comfort; Alpha Med Scientific) and 64-channel MEA chips (MED-P515A; Alpha Med Scientific) coated with Polyethyleneimine (Sigma) and Laminin-511 (Nippi). For culture on MEAs, a φ3.4-mm glass ring was placed in the middle of the MEA probe at the location of the electrode array, and cell suspensions were seeded inside the ring. After 1 h, Neural Maturation basal medium (NM-001-BM100, XCell Science Inc., USA) supplemented with neuron maturation supplement A (NM-001-SA100, XCell Science Inc., USA) and 100 U/mL penicillin/streptomycin (168–23191, Wako) was applied around the ring, and the ring was carefully removed. After 8 days of culture, the medium was replaced with BrainPhys Neuronal medium with SM 1 neuronal supplement (STEMCELL technologies, USA). Human iPSC-derived mature astrocytes (XCL-1 mature astrocytes, AR-001-1V, XCell Science) were seeded at 3.0 × 10^4^ cells per well. Half the media was exchanged every 4 days. QC analysis revealed that hiPSC-derived neurons were >90% β-tubulin III positive and <1% glial fibrillay acid protein (GFAP) positive. QC analysis also revealed that hiPSC-derived astrocytes were >85% GFAP positive astrocytes. Both neurons and astrocytes were of the same hiPS line (XCL-1, XCell Science). All batches satisfied the mentioned QC conditions. Different batches were used between the experiments indicated in Figs 3 and 4 and the experiments in Fig. 5, Supplementary Fig. 3, and Supplementary Fig. 4.

### Immunocytochemistry

Sample cultures were fixed with 4% paraformaldehyde in phosphate-buffered saline (PBS) on ice (4 °C) for 10 min, followed by methanol on ice (−20 °C) for 10 min. Fixed cells were incubated with 0.2% Triton X-100 in PBS for 5 min, followed by preblock buffer (0.05% Triton X and 5% goat serum in PBS) at 4 °C for 1 h, and finally with preblock buffer containing a specific primary antibody (1:1000) at 4 °C for 24 h. The primary antibodies used were rabbit anti-β -tubulin III (T2200, Sigma–Aldrich) for the specific labeling of neurons, goat anti-GFAP (ab53554, Abcam) and mouse anti-Synaptophysin (MAB329, Millipore). Immunolabeling was visualized by incubation in an appropriate secondary antibody anti-rabbit 488 Alexa Fluor (A21206, Thermo Fisher Scientific), anti-mouse 546 Alexa Fluor (Z25004, Thermo Fisher Scientific), and anti-goat 680 Alexa Fluor (ab175776, Abcam), 1:1000 in preblock buffer) for 1 h at room temperature. Cell nuclei were counterstained using 1 μg/mL Hoechst 33258 (H341, DOJINDO) for 1 h at room temperature. Stained cultures were washed twice in preblock buffer (5 min/wash), rinsed twice using PBS, and viewed using a confocal microscope (TCS SP8, Leica). Image intensity was adjusted using ImageJ software (NIH).

### Extracellular recording

Spontaneous extracellular field potentials were acquired at 37 °C under a 5% CO_2_ atmosphere using a 24-well MEA system (Presto; Alpha Med Scientific) and a 64-channel MEA system (MED64-Basic; Alpha Med Scientific) at a sampling rate of 20 kHz/channel. Signals were high-pass filtered at 1 Hz and stored on a personal computer. Spontaneous firing was recorded every week for up to 72 weeks. The spikes in the acquired data were detected using the 100 Hz high-pass filter.

### Pharmacological tests

Spontaneous firing was recorded every week for up to 10 weeks. Spontaneous recordings were obtained for 10 min before treatment and again after the addition of one of the following receptor antagonists to the culture medium: NMDA receptor antagonist D-(-)-2-amino-5-phosphonopentanoic acid (AP-5, 25 μM; ab120003, Abcam) and AMPA/kainate receptor antagonist 6-cyano-7-nitroquinoxaline-2,3-dione (CNQX, 30 μM; C239-5MG, Sigma–Aldrich). The cultures were kept at 37 °C under a 5% CO_2_ atmosphere between recordings and drug administration. To investigate whether hiPSC-derived cortical neurons can generate epileptiform activity, we administered 1,5-pentamethylenetetrazole (PTZ, 1 µM to 1 mM; P0046, Tokyo Chemical Industry Co) and after 4-aminopryridine (4-AP, 1 µM to 60 µM; 275875, Sigma–Aldrich). Spontaneous firing was recorded for 10 min at each concentration. Then, to evaluate the effects of anti-epilepsy drugs, phenytoin (100 µM to 300 µM, 166–12082, Wako) was added to the medium.

### Burst analysis

Electrophysiological activity was first analyzed using Presto and Mobius software (Alpha Med Scientific) and MATLAB. A spike was counted when the extracellularly recorded signal exceeded a threshold of ±5 σ, where σ was the standard deviation of the baseline noise during quiescent periods. SBFs were detected using the 4-step method, which was described previously^45^. Firstly, if the inter-spike interval was within 5–15 ms, these spikes were defined to the same SBF. Secondly, if the maximum of the spikes in the SBF was under 50–100 spikes/SBF, these data sets were eliminated from the SBF. Thirdly, if the inter-SBF interval was under 100–200 ms, these SBFs were combined. Finally, if the summation of spikes during a SBF was over 500–1500 spikes/SBF, we defined the SBF. Appropriate numerical values that can accurately detect bursts with 16 electrodes or 64 electrodes were used as parameter numerical values. All data are expressed as the mean ± standard error (S.E.).

### Frequency analysis

Frequency components above 250 Hz in LFP raw data were removed by a band pass finite impulse response (FIR) filter from 0.1 to 250 Hz using Signal Processing Toolbox in MatLab that were run forward and in the reverse direction and, thus, without phase distortions. Wavelet analyses of LFP were performed using a custom-written program in MatLab (using function cwt () in package “Wavelet Toolbox”). Briefly, the LFP recording, $f(t)$, was transformed as follows:

$${\rm{W}}({\rm{b}},{\rm{a}})=\frac{1}{\sqrt{a}}{\int }_{-\infty }^{\infty }f(t)G(\frac{t-b}{a})dt$$

where a, b denoted the scaling factor (1/Hz) and the center location (ms) of the mother wavelet function, respectively. 1/a varied from 0.1 to 250 Hz.

In Equation (1), $G(x)$ is the complex Morlet function:

$$G(x)=\frac{1}{\sqrt{\pi {F}_{B}}}\exp (-\frac{{x}^{2}}{{F}_{B}})\exp (2i{\rm{\pi }}{F}_{C}x)$$

where ${F}_{B}=5$ was the frequency bandwidth or wavenumber, and ${F}_{C}=1$ was the center frequency.

The wavelet power spectrum (WPS), $|{\rm{W}}({\rm{b}},{\rm{a}})|$, from each LFP recording is shown. The amplitude of this transform was obtained from its absolute value and color-coded. A scalogram is drawn with the Y axis representing the frequency band as 170 pixels and the X axis representing time. One pixel on the X axis is 50 μs, and the number of pixels on the X axis varies depending on the duration of SBF.

To compare the intensity change of the frequency band caused by drug administration, first, a scalogram was created by subtracting the scalogram before and after drug administration. Next, normalization was performed by the following equation. This is because the number of pixels on the Y axis varies depending on the frequency band and the number of pixels on the X axis differs depending on each SBF. The start and end of the SBF were detected using the 4-step method.

$$W{T}_{A}=\frac{W{T}_{S}}{{N}_{X}\,\times \,{N}_{Y}(f)}$$

$W{T}_{A}$: Wavelet transform coefficient per pixel in each frequency band

$W{T}_{S}$: Summation of wavelet transform coefficient in each frequency band

${N}_{X}$: Number of pixels on X axis

${N}_{Y}(f)$: Number of pixels on Y axis, $f$ is the frequency band

Regarding the frequency bands, θ wave (5–8 Hz), α wave (8–14 Hz), β wave (15–25 Hz), γ wave (30–50 Hz), high-γ wave (70–150 Hz), and 150–200 Hz bands were analyzed.

### Statistical analysis

The differences in S/N ratios between the neurons and co-culture samples were evaluated using two-tailed paired Student’s *t*-test. Two-way and one-way ANOVA followed by the Holm–Bonferroni method and Dunnett’s test were used for analyzing other data.

## Electronic supplementary material

Supplementary info

Supplementary movie1
