# Supplementary material for: Toxicological evaluation of convulsant and anticonvulsant drugs in human induced pluripotent stem cell-derived cortical neuronal networks using an MEA system
Source: Sci Rep. 2018 Jul 10;8:10416. doi: 10.1038/s41598-018-28835-7 (PMC6039442; doi:10.1038/s41598-018-28835-7)
Supplement: Supplementary file 1 — Supplementary info [file 41598_2018_28835_MOESM1_ESM.pdf]

Supplementary information

Toxicological evaluation of convulsant and anticonvulsant drugs in human induced pluripotent stem cell-derived cortical neuronal networks using an MEA system

A. Odawara[a, d, e], N. Matsuda[a], Y. Ishibashi[a], R. Yokoi[a], I. Suzuki[a, b, c*]

[a] Department of Electronics, Graduate School of Engineering, Tohoku Institute of Technology, 35-1 Yagiyama Kasumicho, Taihaku-ku, Sendai, Miyagi, 982-8577, Japan

[b] iPS-non Clinical Experiments for Nervous System (iNCENS) Project, Japan

[c] Consortium for Safety Assessment using Human iPS Cells (CSAHi), Japan

[d] Advanced Institute for Materials Research, Tohoku University, 2-1-1 Katahira, Aoba-ku, Sendai, Miyagi, 982-8577, Japan

[e] Japan Society for the Promotion of Science

[*]Corresponding author:

Ikuro Suzuki

Tel: +81-22-305-3219

Fax: +81-22-305-3219

E-mail: i-suzuki@tohtech.ac.jp

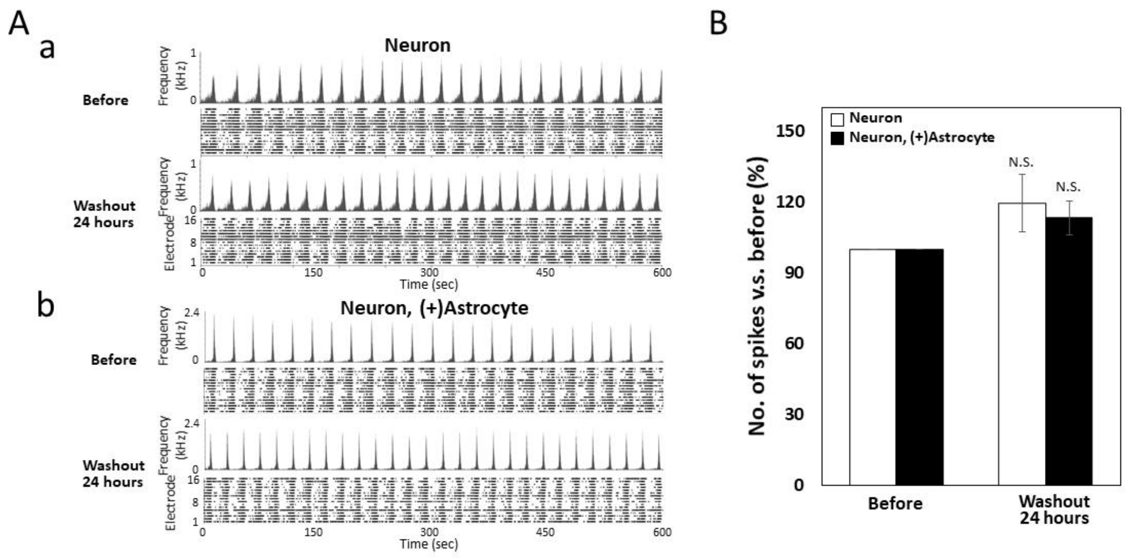

Supplementary Fig. 1

Comparison of spontaneous firings between before drug administration and after 24 hours of drug washout. (A) Raster plots and the histogram of spikes for 10 min before AP-5 (25 μM) administration and after 24 hours of AP-5 and CNQX (30 μM) washout in the neuron only sample (a) and the co-culture sample (b). (B) Comparison of number of spikes before drug administration and after 24 hours of drug washout (n > 4 wells, two-tailed paired Student's *t*-test, N.S. is not significant). Firing rate and burst firings in the neural network were recovered after drug washout.

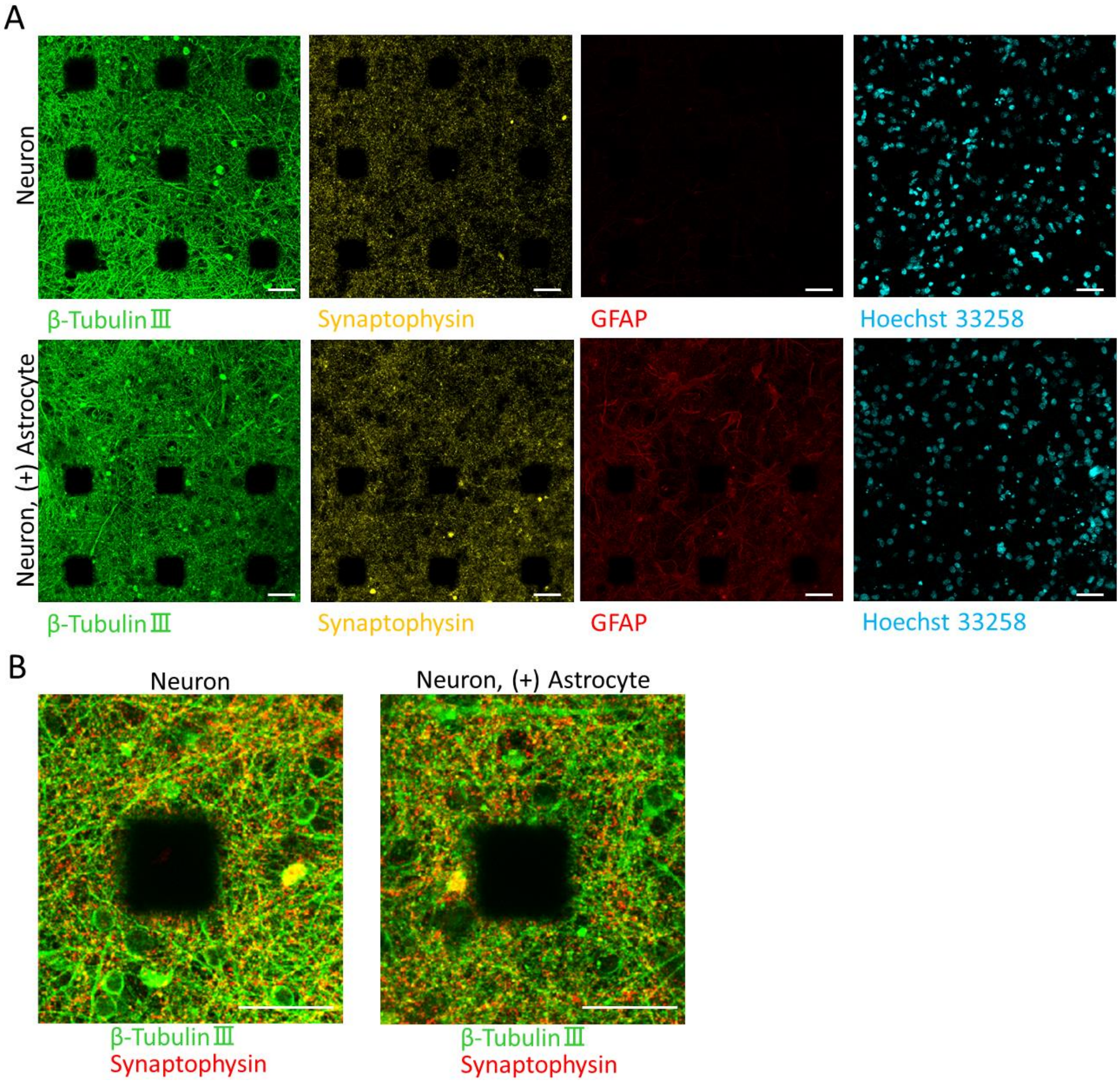

Supplementary Fig. 2

(A) Images show the formation of neuronal networks and synapses in cultured hiPSC-derived neurons on the MEA (upper) and co-culture sample (under) at 11 WIV. Green: neuronal marker β-tubulin III. Yellow: presynaptic marker synaptophysin. Red: astrocyte marker GFAP. Blue: nuclear marker Hoechst 33258. (B) Magnified images show synaptogenesis in cultured hiPSC-derived neurons and co-culture sample. Red: presynaptic marker synaptophysin. Scale bars = 50 μm.

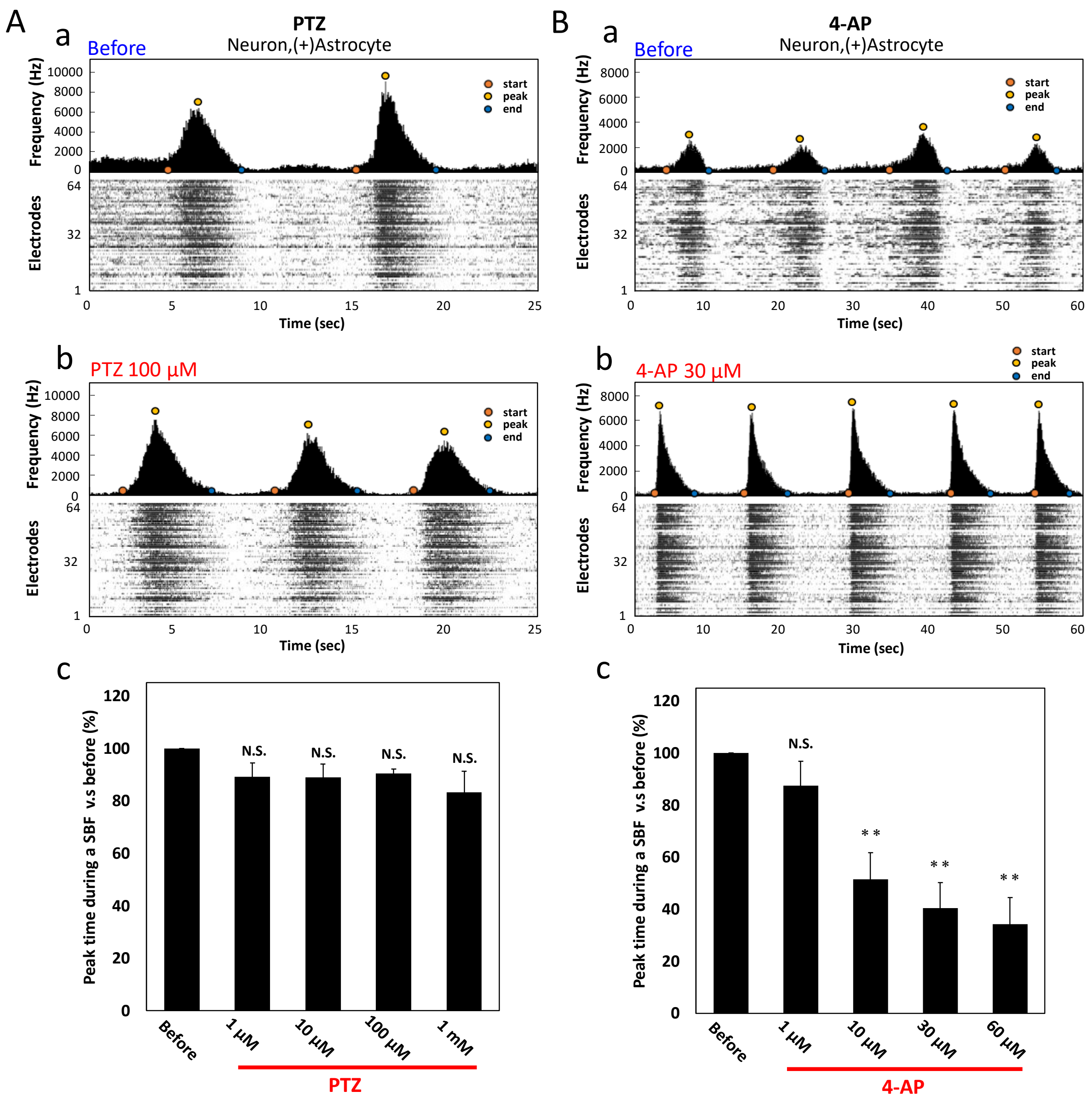

Supplementary Fig.3

The peak time during an SBF with PTZ and 4-AP administration obtained at 64 electrodes per well in co-culture samples. (A) Representative peak of an SBF after PTZ administration. (a) Upper graph shows the histogram of spikes before administration during an SBF obtained at 64 electrodes (orange circle; start time of SBFs, yellow circle; peak time of SBFs, blue circle; end time of SBFs). (b) Under graph shows the histogram of spikes at 100 µM PTZ

administration during a SBF. (c) Dose dependency (n ≧ 3 wells, N.S. is not significant versus before). (B) Representative peak of an SBF with 4-AP administration. (a) Upper graph shows the histogram of spikes before administration during an SBF obtained at 64 electrodes (orange circle; start time of SBFs, yellow circle; peak time of SBFs, blue circle; end time of SBFs). (b) Under graph shows the histogram of spikes during an SBF at 30 μM 4-AP administration. (c) Dose dependency (n ≧ 3 wells, *p < 0.05, **p < 0.01).

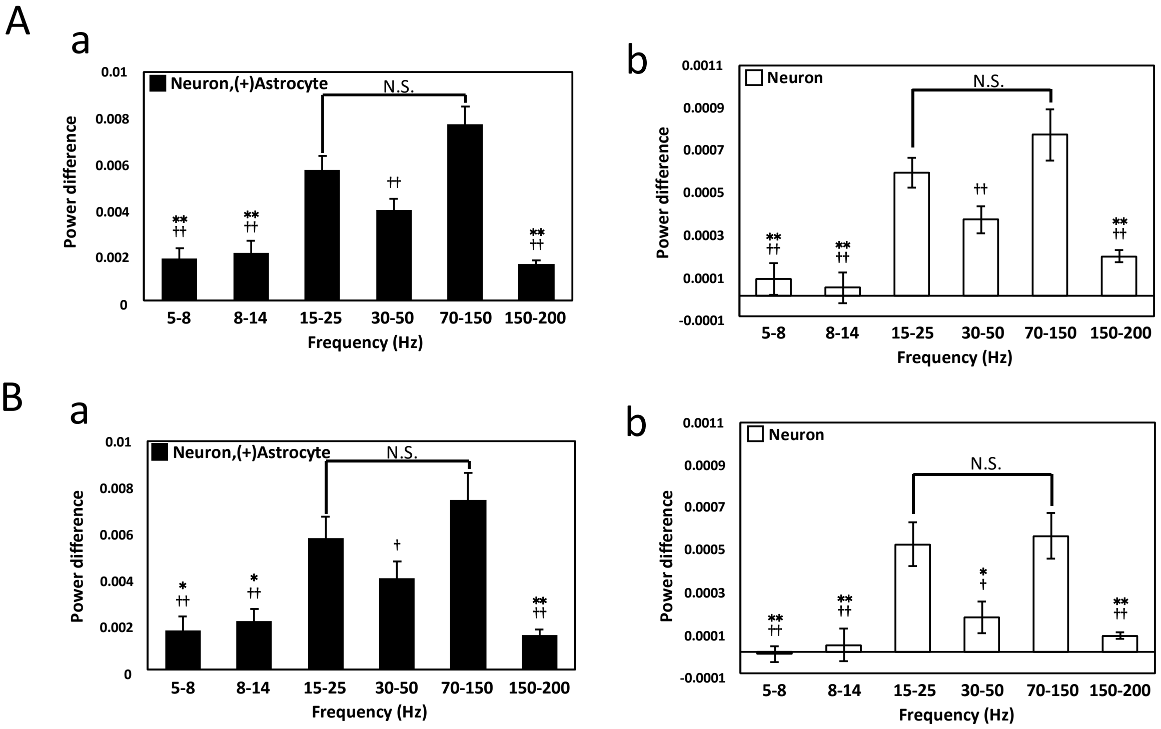

Supplementary Fig.4

The change of low frequency band in PTZ and 4-AP administration. The intensity changes in each band were quantified by average wavelet transform coefficient per pixel in the neurons and co-culture samples. N is the analysis data of five electrodes. Five SBFs per electrode were analyzed, and average values were used. The frequency components of the β wave and the high γ wave were intensified compared with other frequency bands in both

neurons and co-culture samples. The enhancement of frequency components in the co-culture sample was larger than that in the neurons sample. The Holm–Bonferroni method was used for statistical analysis. *indicates a significant difference with respect to β wave band (15–25 Hz), and †indicates a significant difference with respect to high-γ wave band (70–150 Hz). (*$p < 0.05$, **$p < 0.01$, $^{\dagger}p < 0.05$, $^{\dagger\dagger}p < 0.01$) (A) Intensity changes in each band before and after administration of 1 mM PTZ. (B) The intensity changes in each band before and after administration of 30 µM 4-AP.

Supplementary Movie 1

Raw data of spikes in co-culture with astrocytes at 9 WIV before drug administration, after 60 µM 4-AP, and after 100 µM phenytoin. Movie played at 4 ×speed.
